# Substituting ryegrass-based pasture with graded levels of forage rape in the diet of lambs decreases methane emissions and increases propionate, succinate, and primary alcohols in the rumen

**DOI:** 10.1093/jas/skac223

**Published:** 2022-06-20

**Authors:** Maria M Della Rosa, Edgar Sandoval, Peter Reid, Dongwen Luo, David Pacheco, Peter H Janssen, Arjan Jonker

**Affiliations:** Grasslands Research Centre, AgResearch Ltd, Palmerston North, New Zealand; Grasslands Research Centre, AgResearch Ltd, Palmerston North, New Zealand; Grasslands Research Centre, AgResearch Ltd, Palmerston North, New Zealand; Grasslands Research Centre, AgResearch Ltd, Palmerston North, New Zealand; Grasslands Research Centre, AgResearch Ltd, Palmerston North, New Zealand; Grasslands Research Centre, AgResearch Ltd, Palmerston North, New Zealand; Grasslands Research Centre, AgResearch Ltd, Palmerston North, New Zealand

**Keywords:** brassica, ethanol, methane yield, methanol, succinate, volatile fatty acids

## Abstract

Feeding 100% forage rape to sheep consistently lowers methane emissions per unit of intake (CH_4_/DMI) compared to those fed 100% ryegrass pasture. However, forage rape is usually supplemented with other feeds, which might impact the mitigation potential provided by forage rape. The objective of this study was to determine the effect of substituting ryegrass with graded levels of forage rape in the diet of lambs on methane emissions and rumen fermentation characteristics. Seventy wether lambs (*n* = 14/treatment) were fed a ryegrass-based pasture substituted with 0%, 25%, 50%, 75%, and 100% of forage rape (*Brassica napus*; FR0, FR25, FR50, FR75, and FR100, respectively) on a dry matter basis. Methane emissions and dry matter intake were measured for 48 h in respiration chambers and a rumen fluid sample was collected. CH_4_/DMI decreased (*P <* 0.01) with increasing forage rape inclusion in the diet so that sheep fed FR100 and FR75 emitted 34% and 11% less, respectively, than those fed FR0. CH_4_/DMI differences for lambs fed FR25 and FR50 were much smaller (<6%) relative to FR0. The pH of rumen fluid decreased (*P <* 0.01) at higher levels of forage rape inclusion in the diet (FR75 and FR100) compared to low levels of inclusion (FR0, F25, and F50). The proportion of ruminal acetate was least in FR100 (30%) followed by FR75 (10%), FR50 (8%), and FR25 (4%) compared with FR0 (*P <* 0.001). The proportion of propionate plus succinate was greater for FR100 (+40%), FR75 (+28%), and FR50 (+29%) compared with FR0, with FR25 intermediate (*P <* 0.001). The methanol concentration, and ethanol and propanol proportions in rumen fluid were greater for FR100 compared with any other treatment (*P <* 0.001). In conclusion, CH_4_/DMI decreased at high levels of forage rape inclusion in the diet and especially feeding FR100 was associated with a pronounced shift in rumen fermentation profile, with a significant presence of succinate, ethanol, propanol, methanol, valerate, and caproate.

## Teaser

A ration solely comprising forage rape reduces methane emissions per unit of dry matter intake. There is a nonlinear increased reduction of methane with greater levels of forage rape in the diet.

## Introduction

Enteric methane (CH_4_) from ruminants contributes approximately 40% of the greenhouse gas emissions from livestock production (Herrero et al., 2016). A few options for mitigation are available for grazing systems (Leahy et al. 2019), but feeding diets of 100% forage rape (*Brassica napus*) has been postulated as an option for grazing ruminants. Compared to feeding pastures dominated by perennial ryegrass (*Lolium perenne*), feeding 100% forage rape has consistently resulted in less CH_4_ produced per unit of dry matter intake (CH_4_/DMI) in sheep (Sun et al., 2012, 2015a, 2020). However, livestock grazing forage rape are usually supplemented with ryegrass silage or other forages to increase the fiber content of the total diet. Only one study tested the effect of substituting ryegrass-based pasture with graded levels of forage rape in the diet of sheep and CH_4_/DMI decreased linearly with increasing forage rape in the diet at a rate of a 16% reduction for each 25% increase of forage rape in the diet (Sun et al., 2015b). This reduction was unexpected because replacing forage with a more digestible feed (concentrates) in the diet of ruminants usually results in a nonlinear decrease in CH_4_/DMI, with major reductions only at high concentrate inclusion levels (Moss et al., 1995, Sauvant and Noziere 2016, Jonker et al., 2016). Furthermore, Sun et al. (2015a, 2020) hypothesized that the effect of forage rape on CH_4_/DMI was, at least partially, explained by a decreased rumen fluid pH when feeding forage rape compared to those fed ryegrass. However, one would expect the rumen fluid pH to substantially drop only when a threshold level of rapidly fermentable dietary carbohydrates in the diet is exceeded (Zebeli et al., 2012), and therefore, the change in rumen liquid pH would be expected to change nonlinearly with increasing fermentable dietary carbohydrates in the diet.

Forage rape has greater digestibility than perennial ryegrass and is also more readily fermentable and has less structural carbohydrates, which are thought to be responsible for a rapid degradation rate of forage rape and result in a lower mean rumen fluid pH (Sun et al., 2015a). Methanogens are sensitive to pH, and pH < 6 inhibits CH_4_ production (Van Kessel and Russell, 1996; Russell, 1998). If CH_4_ production decreases, dissolved H_2_ gas concentrations in the rumen liquid increase, driving changes in rumen fermentation pathways (Janssen, 2010). As a result, acetate production and H_2_ release associated with acetate formation both decrease, whereas synthesis of propionate and other electron-accepting fermentation products increases in importance as electron sinks (Janssen, 2010; Ungerfeld, 2015). However, the response in changes in rumen fermentation products with increasing levels of forage rape has been limited mostly to the study of the “major” (acetate, propionate, and butyrate) with some studies also reporting “minor” (e.g., valerate, iso-butyrate, iso-valerate, and caproate) organic acids. A fuller metabolic picture is required to better understand the rumen processes when methanogenesis is reduced (Leahy et al., 2022).

The hypothesis of this study was that increasing the substitution of perennial ryegrass-based pasture with forage rape in the diet of sheep would result in a linear reduction in CH_4_/DMI. If this would be confirmed, then forage rape could be used over a wide range of inclusion levels to mitigate methane emissions. Therefore, in the present study, ryegrass-based pasture was substituted with graded levels of forage rape (0%, 25%, 50%, 75%, and 100% of diet) and the ruminal fermentation metabolites (including seldomly reported fermentation intermediates and alcohols) were related to changes in CH_4_/DMI.

## Materials and Methods

The present experiment determined CH_4_ emissions from 10-mo-old wether lambs fed fresh perennial ryegrass-based pasture (*Lolium perenne; cv* Ceres 150, referred to as ‘ryegrass’ from here on) substituted with graded levels of winter forage rape (*Brassica napus; cv* Titan). The animal experiment adhered to the guidelines of the 1999 New Zealand Animal Welfare Act and the AgResearch Code of Ethical Conduct. Animal manipulations were approved by the AgResearch Grasslands Animal Ethics Committee (Palmerston North, approval number 14990).

### General management and treatments

One hundred lambs (42 ± 0.4 kg body weight, mean ± SD) were randomly assigned to one of five dietary treatments (percentages on dry matter basis): 100% ryegrass (FR0), 75% ryegrass + 25% forage rape (FR25), 50% ryegrass + 50% forage rape (FR50), 25% ryegrass + 75% forage rape (FR75), and 100% forage rape (FR100). The lambs assigned to the four treatments with forage rape were transitioned and adapted to grazing forage rape for 3 wk as one group at the AgResearch Aorangi farm (near Palmerston North, New Zealand), whereas the fifth group (FR0) continued grazing pasture on the same farm. After this diet transition and adaptation phase of 21 d, 16 lambs were selected from each treatment, based on health status and mid-range of body weight change during the adaptation period. These 80 lambs were transported to the Grasslands Research Centre (AgResearch Ltd., Palmerston North, New Zealand) for adaptation to indoor housing and feeding of cut forages, followed by a measurement phase (Supplementary Table S1).

At AgResearch Grasslands, animals were housed by treatment in 10 group pens with 8 animals per pen (two pens per treatment). The lambs were fed their respective full diets for at least 14 d before the start of the measurement period. Diets were delivered twice daily in two equal portions at around 0830 and 1530 h. Feed was offered at approximately 1.8 times maintenance energy requirements calculated according to CSIRO (2007), assuming 11.5 MJ metabolizable energy per kg of dry matter for both forages. It was assumed that sheep would not eat the main stem of forage rape (as observed in the field and in other trials) and therefore the forage rape offer was increased to account for this. The lambs had *ad libitum* access to water.

### CH_4_ measurements

Before CH_4_ measurements in respiration chambers, animals were in individual crates for two days for adaptation, and then, 14 lambs from each treatment group were selected for CH_4_ measurements in respiration chambers for 2 d. The CH_4_ measurements were performed at the New Zealand Ruminant Methane Measurement Centre (AgResearch Ltd., Palmerston North, New Zealand), which comprises three clusters with eight open-circuit respiration chambers each (24 chambers). The 70 animals were grouped into three batches for the CH_4_ measurements. Because 24 respiration chambers were available and there were five treatments, it was not possible to balance the number of animals per treatment per measurement batch. Therefore, gas emissions were measured in five animals from four treatments and four animals from one treatment in the first and the second batch, whereas four animals from three treatments and five animals from two treatments were in the third batch. The three batches went into chambers in three consecutive weeks.

The respiration chambers system used was previously described in detail (Pinares-Patiño et al., 2012). Briefly, each chamber was 1.84 m^3^ (1.8 m long × 0.85 m wide × 1.2 m high) with an airflow rate of around 300 L/min, which was continuously monitored by measuring differential pressure within a Venturi flowmeter. The temperature inside the respiration chambers was approximately 14.5 °C and the relative humidity was on average approximately 72%. Subsamples of outflow gases from each of eight chambers in a cluster were continuously drawn to a multiport gas switching unit (S.W. & W.S. Burrage, Ashford, Kent, UK). The air stream of each individual chamber was directed in sequence (over 5- to 6-min intervals) to a 4900C Continuous Emissions Analyzer (Servomex Group, Brighton, East Sussex, UK; one analyzer per cluster) to determine CH_4_ and carbon dioxide (CO_2_) concentrations by infrared technology and H_2_ concentrations by paramagnetic cell. The analysers were tested daily using alpha-standards (BOC, Auckland, New Zealand) and calibrated if required (Pinares-Patiño et al., 2012). The recovery of CH_4_ from each chamber through the whole system was independently tested by the National Institute of Water and Atmospheric Research (Wellington, New Zealand) approximately 6 mo before this study, and the mean CH_4_ recovery percentage was 95 ± 8% (mean ± SD).

All chambers were opened twice daily at around 0830 and 1530 h (front and rear doors of each chamber) for approximately 30 min to allow the collection of feed refusals, provide fresh feed, refill water bins, and exchange excreta collection trays for clean ones. Gas emissions during these periods were extrapolated by taking the average of the last 12 values before opening the door.

### Forage offered

Forage fed to animals during the indoor diet adaptation period and measurement period was harvested daily at around 1030 h at Aorangi farm during July–August 2020. Forage rape and ryegrass were both at a vegetative growth stage. The harvested forage was weighed into two equal meals for feeding at approximately 1530 h and the second portion was stored at 4 °C for feeding the next morning. A sub-sample of each forage was collected daily for dry matter (DM) determination by oven drying at 105 °C for 24 h in triplicate. During days in crates and chambers, a second sub-sample was frozen at −20 °C for later chemical analysis. For forage rape, a third sub-sample was collected for determination of plant part proportions and dry matter of each part (triplicate subsamples), including lamina, petiole, main stem, and dead leaves (Jian et al., 2017). Each part was dried at 65 °C to constant weight. Harvested forage rape offered during the measurement period consisted on average on a dry matter weight basis of 42% green lamina, 27% petiole, 4% dead leaves, and 27% main stems.

### Forage analysis

Before chemical analyses, forages fed within each measuring batch were pooled across 2 d in crates and 2 d in respiration chambers. Within each measuring batch, each forage rape plant part was pooled in equal parts across four measurement days (2 d in crates and 2 d in chambers).

Samples of ryegrass and whole forage rape were freeze-dried and ground to pass a 1-mm screen (Model 4; Thomas Wiley, Philadelphia, PA). Samples were analyzed for ash (AOAC 942.05), crude protein (CP; N × 6.25, AOAC 968.06), and crude fat (Cfat; AOAC 930.15) and minerals following AOAC (1990) procedures. Neutral detergent fiber (NDF), acid detergent fiber (ADF), and lignin were analyzed according to Van Soest et al. (1991) and are expressed inclusive of residual ash. Soluble sugars were extracted using ethanol:water (80:20) solution and quantified by the anthrone colorimetric method (Yemm and Willis, 1954). Homogalacturonan was quantified and defined as pectin (Blumenkrantz and Asboe-Hansen, 1973). The concentrations of nitrate-N in forages were quantified using the method described by Cataldo et al. (1975). Nonfiber carbohydrates (NFC) were calculated as 100 − (CP + ash + Cfat + NDF). The concentration of cobalt in forage samples was quantified by inductively coupled plasma mass spectrometry after nitric acid/hydrogen peroxide digestion (Huang et al., 2004). Results were calculated according to the percentage of ryegrass and forage rape included in each treatment (Table 1).

**Table 1.** Mean ± standard deviation of dry matter and chemical composition of perennial ryegrass and forage rape fed to sheep during methane emission measurements in respiration chambers

Nutrient profile^2^	Forage rape inclusion level^1^				
	FR0	FR25	FR50	FR75	FR100
Dry matter, DM, g/kg	145 ± 15	149 ± 6	153 ± 9	157 ± 6	162 ± 4
Ash, g/ kg DM	117 ± 3	108 ± 3	99 ± 3	89 ± 3	81 ± 3
Organic matter profile, g/kg DM					
Crude protein	233 ± 11	211 ± 9	188 ± 6	164 ± 3	143 ± 4
Crude fat	41 ± 3	39 ± 2	38 ± 1	36 ± 1	34 ± 1
NFC	175 ± 24	274 ± 19	375 ± 17	471 ± 14	571 ± 9
Soluble sugars	89 ± 17	145 ± 15	202 ± 15	256 ± 13	312 ± 11
Neutral detergent fiber, NDF	439 ± 13	371 ± 14	302 ± 13	235 ± 12	169 ± 4
Acid detergent fiber	236 ± 5	207 ± 5	179 ± 5	150 ± 6	123 ± 4
Lignin	23 ± 3	29 ± 6	35 ± 10	41 ± 15	46 ± 20
Nitrate-N, mg/kg DM	618 ± 155	485 ± 107	362 ± 63	230 ± 29	102 ± 3
Pectin	10 ± 0.6	22 ± 1.2	34 ± 1.6	46 ± 0.3	58 ± 0.2
iNDF	94 ± 2.7	75 ± 2.0	56 ± 2.7	37 ± 2.5	19 ± 6.8
pdNDF	338 ± 16.1	291 ± 12.5	243 ± 10.2	196 ± 10.9	150 ± 4.9
Mineral profile, g/kg DM					
Phosphorus	5.0 ± 0.18	4.6 ± 0.14	4.2 ± 0.08	3.8 ± 0.02	3.5 ± 0.11
Potassium	44.1 ± 1.31	38.2 ± 1.07	32.2 ± 0.85	26.4 ± 0.70	20.7 ± 0.47
Sulfur	4.1 ± 0.04	4.5 ± 0.05	4.9 ± 0.08	5.2 ± 0.02	5.6 ± 0.11
Calcium	5.2 ± 0.14	7.4 ± 0.21	9.8 ± 0.35	11.9 ± 0.33	14.3 ± 0.51
Magnesium	2.1 ± 0.05	2.1 ± 0.02	2.2 ± 0.01	2.2 ± 0.04	2.2 ± 0.06
Sodium	1.2 ± 0.06	1.0 ± 0.06	0.8 ± 0.06	0.6 ± 0.05	0.4 ± 0.05
Chloride	20.3 ± 0.84	18.4 ± 0.66	16.5 ± 0.47	14.6 ± 0.26	12.8 ± 0.44
Cobalt, µg/kg DM	36.1 ± 0.01	31.7 ± 0.47	27.4 ± 0.61	23.0 ± 0.96	18.9 ± 0.01

FR0, 100% ryegrass; FR25, 75% ryegrass + 25% forage rape; FR50, 50% ryegrass +50% forage rape; FR75, 25% ryegrass + 75% forage rape; FR100, 100% forage rape.

NFC, Non-fiber carbohydrates, calculated as 100 − (crude protein + ash + crude fat + neutral detergent fibre); iNDF, indigestible neutral detergent fiber; pdNDF, potentially digestible NDF, calculated as NDF − iNDF.

### Body weight, dry matter intake, nutrients intake

Body weight was recorded each ~5–7 d during the diet adaptation period on-farm. Weights were recorded around 0900 h, before animals got access to a new strip of forage to graze. Animals were weighed at arrival at Grasslands and at 7 and 14 d after arrival during the indoor adaptation period. Animal body weights were also recorded before entering individual crates and after leaving the respiration chambers (Table 2).

**Table 2.** Mean dry matter intake (DMI), estimated total tract dry matter digestibility (DMD) and methane (CH_4_), carbon dioxide (CO_2_), and hydrogen (H_2_) emissions from sheep fed ryegrass-based pasture substituted with increasing levels of forage rape

Parameter	Forage rape inclusion level^1^					SED	*P*-value	linear	quadratic
	FR0	FR25	FR50	FR75	FR100				
DMI, kg/d	0.98^c^	1.01^bc^	1.08^ab^	1.10^a^	1.06^abc^	0.03	<0.01	0.02	0.08
DMD, g/kg DM	787^a^	842^b^	874^c^	919^d^	961^e^	8.20	<0.01	0.01	0.55
CH_4_, g/d	18.5^a^	18.9^a^	19.3^a^	18.4^a^	13.2^b^	0.71	<0.01	<0.01	<0.01
CH_4_, g/ kg DMI	19.2^a^	18.7^a^	18.1^ab^	17.0^b^	12.7^c^	0.66	<0.01	<0.01	<0.01
CH_4_, g/kg dDMI^2^	25.1^a^	21.5^b^	21.1^b^	18.5^c^	13.3^d^	1.13	<0.01	<0.01	0.07
CH_4_/CO_2_, mol/mol^3^	0.054^a^	0.054^a^	0.052^ab^	0.049^b^	0.036^c^	0.002	<0.01	<0.01	<0.01
CO_2_, g/kg DMI	963.5	957.7	953.7	969.9	964.7	29.8	0.98	0.93	0.64
H_2_, g/kg DMI ^4^	0.05^c^	0.08^bc^	0.12^b^	0.12^b^	0.37^a^	0.11	<0.01	<0.01	<0.01

FR0, 100% ryegrass; FR25, 75% ryegrass + 25% forage rape; FR50, 50% ryegrass +50% forage rape; FR75, 25% ryegrass + 75% forage rape; FR100, 100% forage rape; dDMI, digested dry matter intake; DM, dry matter. SED, average standard error of differences of means.

dDMI, digested dry matter intake.

Calculated as (mol CH_4_/d)/(mol CO_2_/d).

Log-transformed variable for statistical analysis, reported values were back-transformed.

Treatment means within a column with different superscripts are significantly different (*P <* 0.05).

Dry matter intake (DMI) was measured for 2 d in individual crates and 2 d in respiration chambers. The DMI was calculated as the difference between the dry matter of feed offered and refused. Feed refusals were collected once a day before morning feeding. The total weight of fresh refusals was recorded and then forage rape stems were separated from the refusals and their fresh weight was also recorded. The DM content of refusals from each individual animal, excluding the main forage rape stem, i.e., edible refusals, was quantified by oven drying a sample at 65 °C to a constant weight. The main stems were pooled per day across animals and triplicate sub-samples were dried at 65 °C to a constant weight. The DM refused per sheep was calculated as DM% of edible refusals for each individual animal multiplied by the fresh matter in edible refusals for each individual animal, plus the DM% of stems of the pooled refusals multiplied by the fresh matter of stems in refusals recorded for each individual animal. Each single main stem of forage rape is quite long and thick and has a considerable weight. Therefore, it is impossible to collect a representative sample of refusals if stems were present. For this reason, stems were dried separately from the rest of the refusal to ensure the DM determination was not biased by the DM content of stems vs. the rest of the refusals.

### Collection of fecal samples and estimation of dry matter digestibility

Fecal spot-samples were collected to determine indigestible neutral detergent fiber (iNDF) as an internal marker to estimate total tract dry matter digestibility (DMD). The fecal samples were collected from waste collection trays just before morning and afternoon feeding during 2 d in individual crates and 2 d in respiration chambers. Samples were collected from a feces-collection-mesh with 5-mm holes to keep feces separated from urine. Equal aliquots of the eight spot-samples per sheep were pooled in a container and frozen at −20 °C.

Pooled fecal spot-samples collected per animal were freeze-dried and ground to pass a 1-mm screen. Then, iNDF was quantified in forage offered and fecal samples according to Huhtanen et al. (1994). Briefly, samples were incubated in situ in filter bags for 12 d in the rumen of two cannulated cows fed a ryegrass-based pasture ad libitum and supplemented with ryegrass hay. Samples were incubated in six F57 filter bags (Ankom Technology, Macedon, NY) with 0.5 g of DM for each sample, and six empty filter bags were also incubated as blanks. After ruminal incubation, filter bags were washed under tap water and dried at 65 °C for 48 h. Then, the NDF of the incubation residue was determined according to the method of Van Soest et al. (1991) and iNDF was calculated. Next, DMD was calculated as 100 × [1 − (iNDF_feed_/iNDF_faeces_)] (Huhtanen, et al., 2007).

### Rumen fluid sampling and analyses

Rumen fluid samples were collected by esophageal-stomach tubing, using a 130-mL syringe coupled to a flexible tube (milk vacuum hose), before the morning feeding, after the lambs were removed from the respiration chambers. The pH of the rumen fluid samples was measured without further processing immediately after collection, using a calibrated pH meter (Orion Star A211; Thermo Fisher Scientific, Waltham, MA). Then, a subsample of 1.8 mL was placed on ice and, when back in the laboratory, this sample was centrifuged at 21,000 × *g* for 10 min at 4 °C. After centrifugation, 900 μL of the supernatant were transferred to a new tube containing 100 μL of internal standard (19.87 mM 2-ethylbutyric acid in 20%, vol/vol phosphoric acid) and stored at −20 °C until analysis of short-chain fatty acids (SCFA; acetate, propionate, butyrate, valerate, caproate, iso-butyrate, and iso-valerate), ammonia, alcohols (methanol, ethanol, and propanol), and fermentation intermediate products (succinate, lactate, and formate).

After at least 24-h storage, samples containing the internal standard were thawed and centrifuged at 21,000 × *g* for 10 min at 4 °C. Molar concentrations of SCFAs, alcohols, and intermediate products were analyzed by gas chromatography. Concentrations of SCFAs and alcohols were determined as described by Tavendale et al. (2005) using a Shimadzu GC-2010 Plus and AOC 20i auto-injector (Shimadzu Corporation, Kyoto, Japan), a Phenomenex Zebron ZB-FFAP Capillary GC Column of 30 m length x 0.53 mm I.D x 1.00 μm film thickness (Phenomenex, Torrence, CA); helium was the carrier gas (BOC, Palmerston North, NZ). The flame ionization detector was set to 240 °C, and the column temperature was 60 °C for 3.5 min, increased to 120 °C at 30°C/min, increased to 185 °C at 10 °C/min, increased to 200 °C at 15 °C/min, and then held at 200 °C for 3 min to separate SCFAs from alcohols.

Intermediate products, i.e., formate, lactate, and succinate, were analyzed according to Richardson et al. (1989). Concentrations of intermediate products were determined using a Shimadzu GC-2010 Plus equipped with a barrier ionization discharge detector, AOC 6000 auto-sampler, VICI heated helium purifier (Valco Instruments, Houston, TX), and a Phenomenex Zebron ZB-5MS Capillary GC Column of 30 m length × 0.25 mm I.D × 0.25 µm film thickness (Phenomenex). Helium was the carrier gas (BOC, Palmerston North, NZ). The barrier ionization discharge detector temperature was set to 250 °C and, the GC column temperature was set to 50 °C for 2 min, then increased to 130 °C (5 °C/min), increased to 240 °C (15 °C/min), and then held at 240 °C for 4.67 min.

The detection ranges for alcohols and intermediate fermentation products were 0.6–6.0 mM methanol, 1.0–5 mM ethanol, 0.2–2.0 mM propanol, 0.4–3.0 mM formate, and 0.9–7.4 lactate, and for succinate the detection range was 0.41–3.38 mM when values were lower than 2.0 mM and 2.1–16.91 mM if the values were greater than 2.0 mM. Alcohol concentrations below the detection range were considered, but their absolute value may be inaccurate. Ammonia was quantified by the phenol hypochlorite reaction method (Weatherburn, 1967) scaled down to read on a spectrophotometer with 96-well plate reader at 625 nm. All fermentation products, excluding methanol, were expressed as a percentage of total organic fermentation products (TOFP) measured. Methanol was not included in TOFP calculation because it was assumed to come from methoxy groups released from galacturonic acid in pectin, not from hexose fermentation.

The location of the fluid sampling might not be consistent with the stomach tubing method, and samples can contain variable quantities of saliva contamination. These factors may affect the absolute pH and concentrations of fermentation metabolites in the rumen fluid, but not of fermentation product percentages and ratios (Larsen et al., 2020), and the relative values of pH and SCFA concentrations were previously found to be useful for ranking CH_4_/DMI in sheep (Jonker et al. 2019).

### Blood sampling and urea analysis

Blood samples were collected just before morning feeding, coinciding with rumen fluid sampling. Around 10 mL of blood was collected via jugular vein-puncture using an evacuated tube containing EDTA (Vacutainer; Beckton-Dickinson, Franklin Lakes, NJ). Back in the laboratory, the samples were centrifuged at 1246 × *g* for 20 min at room temperature. After centrifugation, one subsample of 1.5 mL plasma was stored at −20 °C for urea analysis. Urea was quantified by a colorimetric reaction using the UREA RANDOX kit (Randox Ltd, Crumlin, County Antrim, UK) to read on a spectrophotometer with 96-well plate reader at 340 nm.

### Hydrogen balances

To estimate the theoretical H_2_ available for methanogens to produce CH_4_ by either reduction of CO_2_ or methyl compounds, the balance of electron pairs (equivalent to H_2_) produced and accepted in the fermentation of glucose was first calculated based on rumen TOFP concentrations (Wolin, 1960). This calculation assumes that the ratios of fermentation products in rumen fluid samples were proportional to their production rate, and gives an H_2_ amount in mmol/L equivalent to the SCFA concentrations. Electron pairs are generated in the formation of acetate, butyrate, and caproate, whereas electrons pairs are used to form propanol, succinate, or propionate. Ethanol formation from glucose has an electron pair balance of zero. Methanol was assumed to be derived from methoxyl groups rather than fermentation of the core carbohydrates, and, like CO_2_, a sink for H_2_ rather than a sink or source of electrons, and so was not included in the electron pair balance. Excess electron pairs result in the generation of H_2_, i.e., the available H_2_. The available H_2_ is expressed as a nominal dissolved concentration (mM) and was calculated as


$$\begin{array}{ll} Available\;H_{2}= & [(2\times acetate+2\times butyrate+2\times \\ & caproate+0\times ethanol)\\ & -(3\times propanol+1\times succinate+1\times \\ & propionate+1\times valerate)]. \end{array}$$ (1)

The concentration of glucose equivalents fermented was calculated as the sum of fermentation products multiplied by the moles of each product theoretically produced per mole of glucose:


$$\begin{array}{ll} Glucose\;equivalent\;fermented= & \;0.5\times acetate+0.5\times propionate\\ & +1.0\times butyrate+1.0\times valerate+1.5\\ & \times caproate+0.5\times ethanol+0.5\times \\ & propanol+0.5\times succinate. \end{array}$$ (2)

Then, available H_2_ per unit of glucose equivalent fermented was calculated.

The net oxidation/reduction state of the rumen fermentation products was calculated as the sum of the net oxidation equivalent per mole (number of oxygens − number of H_2_/2) for each fermentation product multiplied by the concentration of that fermentation product (Van Soest, 1994). The net oxidation equivalents per mole were as follows: acetate = 0, propionate = −1, butyrate = −2, valerate = −3, caproate = −4, ethanol = −2, propanol = −3, and succinate = +1. The calculations assumed the metabolites were undissociated acids. Iso-butyrate, iso-valerate, and methanol were excluded because these were assumed not to be formed from carbohydrate fermentation.

### Statistical analysis

The data were analyzed by linear mixed-effects model with treatment (FR0, FR25, FR50, FR75, and FR100) as a fixed effect and measurement batch and chamber nested in a cluster as random effects. H_2_ emission data were natural log-transformed before analysis to achieve model assumptions of normality of the data. SCFAs, alcohols, and intermediate fermentation products were analyzed using the above model, but with batch as random effect only. When heteroscedasticity was identified, as was the case for TOFP, SCFA, alcohols, succinate, and ammonia, the variance was modeled. In the model, the polynomial effect of treatment was checked by a permutation test. A multiple treatment comparison was performed on the modeling results with *P*-value adjusted by the “BH” method (Benjamini and Hochberg, 1995). The analysis was performed using packages “lme4” and “predictmeans” in R (R Core Team, 2021). Significance of mean differences and the linear and quadratic effects were declared at *P <* 0.05, and trends were declared when 0.05 < *P <* 0.10. Spearman correlation and linear and quadratic regressions between CH_4_/DMI or CH_4_/dDMI (g methane/kg of digested dry matter intake) averaged over 2 d per lamb and explanatory variables, i.e., rumen fluid pH, DMD, H_2_ produced per unit of glucose equivalent fermented and oxidate state, were performed to determine the relationship of changes in rumen fermentation parameters with the nonlinear decrease in CH_4_/DMI.

## Results

**Figure 1.**
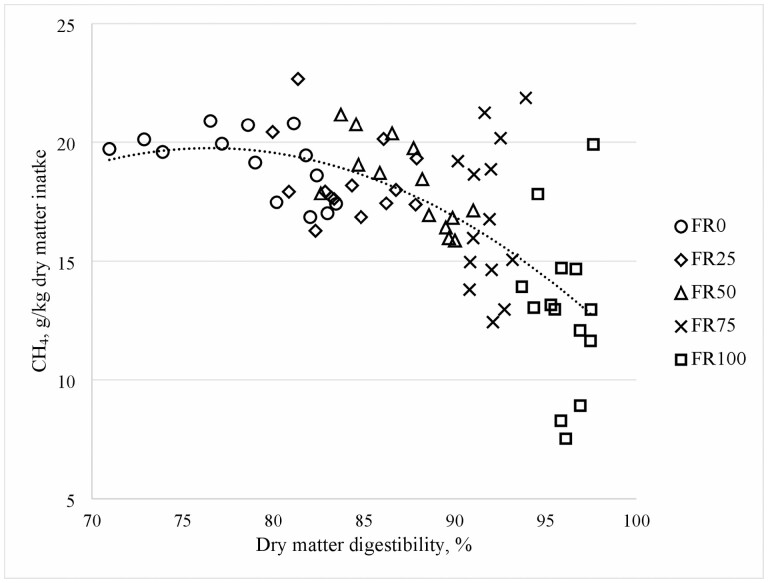
Quadratic relationship between methane (CH_4_) per unit of dry matter intake and dry matter digestibility in sheep fed ryegrass-based pasture substituted with increasing levels of forage rape: FR0: 100% ryegrass, FR25: 75% ryegrass + 25% forage rape, FR50: 50% ryegrass +50% forage rape, FR75: 25% ryegrass + 75% forage rape, FR100: 100% forage rape.

Increasing the proportion of forage rape in the diet of sheep resulted in a non-linear response in CH_4_ production (*P <* 0.01) and CH_4_/DMI (*P <* 0.01) (Table 2). Differences in CH_4_/DMI and other variables were small FR25 and FR50 compared to FR0, but were more pronounced with greater forage rape inclusion for FR75 and FR100. CH_4_ production and CH_4_/DMI were lower (*P <* 0.01) in sheep fed FR100 compared with those fed the other four treatments and sheep fed FR75 had less CH_4_/DMI than those fed FR0 and FR25 (*P <* 0.05), with FR50 being intermediate. The DMD increased linearly (*P <* 0.01) with increasing forage rape inclusion in the diet and also the CH_4_/dDMI decreased linearly (*P <* 0.01) with increasing forage rape. The CH_4_/dDMI was 14% and 16% less in FR25 and FR50 compared with FR0, whereas it was 26% and 47% less in FR75 and FR100, respectively, compared with FR0. The DMD included as linear and quadratic terms in regression explained 46% of the variability in CH_4_/DMI (Figure 1) and was negatively correlated with CH_4_/dDMI (*r* = −0.61, Table 4). H_2_ emitted per unit of DMI was greater in FR100 compared with any other dietary treatment (*P <* 0.01), whereas it was less in FR0 than in FR50 and FR75, with FR25 intermediate. CO_2_ per unit of DMI was similar across dietary treatments, but the molar ratio of CH_4_ to CO_2_ emissions decreased quadratically (*P <* 0.01) with increasing forage rape inclusion in the diet. The molar ratio of CH_4_ to CO_2_ was 9% and 33% less in FR75 and FR100, respectively, compared with FR0, and the ratio was similar for FR0, FR25, and FR50.

**Table 4.** Spearman correlation coefficients between CH_4_ per unit of dry matter intake (CH_4_/DMI) or per unit of digested dry matter intake (CH_4_/dDMI) and estimated dry matter digestibility (DMD), rumen parameters

Parameters^1^	CH_4_/DMI	CH_4_/dDMI	Rumen fluid pH	H_2_/GEF
CH_4_/dDMI	0.92	–	–	–
RumenfluidpH	0.36	0.47	–	–
DMD, g/kg DM	−0.61	−0.80	−0.54	−0.81
H_2_/GEF	0.60	0.75	0.60	–
O/R state	0.34	0.46	0.65	0.65

DM, dry matter; H_2_/GEF, hydrogen produced per unit of glucose equivalent fermented; O/R state, net oxidation reduction state of rumen metabolites (excluding iso-butyrate, iso-valerate, and methanol).

Rumen fluid pH responded nonlinearly (*P <* 0.01) to increasing forage rape inclusion in the diet. Sheep fed FR100 had the lowest rumen fluid pH, and FR75 had a lower rumen fluid pH value than FR0, FR25, and FR50 (Table 4). When rumen fluid pH was included in a quadratic regression equation, it explained 24% of the variability in CH_4_/DMI (−1.2123x^2^ + 19.324x − 57.829) and 31% of the variability in CH_4_/dDMI. pH was positive correlated with CH_4_/DMI (*r* = 0.36) and CH_4_/dDMI (*r* = 0.47) (Table 4).

Most of the individual SCFA proportions (propionate, butyrate, caproate, iso-butyrate, and iso-valerate) changed linearly with increasing forage rape in the diet (*P <* 0.01), whereas acetate (*P <* 0.01) and valerate (*P <* 0.01) proportions decreased nonlinearly with increasing forage rape inclusion in the diet (Table 3). The smallest acetate proportion was in FR100 (−30%) followed by FR75 (−10%), FR50 (−8%), and FR25 (−4%) compared with FR0. The butyrate proportion was greater in FR50, FR75, and FR100 compared with FR0, whereas propionate proportion was greater for any treatment which included forage rape compared with FR0. Formate was detected only in one sample from FR100, whereas lactate was detected in none of the rumen fluid samples. The proportion of succinate increased non-linearly with forage rape inclusion in the diet (*P <* 0.01), being 26 to 385 times greater in FR100 than for any other treatment (Table 3).

**Table 3.** Pre-feeding mean rumen pH, ammonia (NH_4_), short-chain fatty acids, other acids, alcohols, available H_2_ per glucose equivalent fermented, and net oxidation–reduction state of rumen metabolites in sheep fed ryegrass-based pasture substituted with increasing levels of forage rape

Parameter^2^	Forage rape inclusion level^1^					SED	*P*-value	Linear	w
	FR0	FR25	FR50	FR75	FR100				
pH	7.1^a^	7.0^a^	7.0^a^	6.7^b^	6.2^c^	0.13	<0.01	<0.01	<0.01
NH_4_, mM	10.3^a^	7.9^b^	6.2^bc^	4.4^c^	4.2^c^	1.00	<0.01	<0.01	0.07
Blood urea, mM	7.1^a^	6.2^b^	5.8^b^	4.8^c^	4.6 ^c^	0.32	<0.01	<0.01	0.27
TOFP, mM	48.5^d^	52.9^cd^	61.8^bc^	71.8^b^	97.8^a^	6.61	<0.01	<0.01	0.02
SCFA, mM	42.3^d^	52.6^cd^	61.3^bc^	71.3^ab^	85.8^a^	6.69	<0.01	<0.01	0.23
	Short-chain fatty acids, other acids and alcohols, % total organic fermentation acids								
Acetate, A	67.4^a^	64.8^b^	61.9^c^	60.5^c^	47.1^d^	1.52	<0.01	<0.01	<0.01
Butyrate, B	10.0^c^	11.1^bc^	11.8^ab^	13.8^a^	13.5^a^	0.82	<0.01	<0.01	0.54
Propionate, P	16.7^c^	18.8^b^	21.5^a^	21.4^a^	23.4^a^	1.25	<0.01	<0.01	0.47
Caproate	0.25^b^	0.34^b^	0.32^b^	0.52^a^	0.54^a^	0.09	<0.01	<0.01	0.79
Valerate, V	0.97^c^	1.14^bc^	1.07^bc^	1.21^b^	2.08^a^	0.16	<0.01	<0.01	<0.01
Iso-butyrate	1.96^a^	1.58^b^	1.26^c^	0.97^d^	0.35^e^	0.14	<0.01	<0.01	0.26
Iso-valerate	2.26^a^	1.71^b^	1.32^c^	1.03^c^	0.36^d^	0.17	<0.01	<0.01	0.78
Succinate, S	0.03^c^	0.04^c^	0.44^b^	0.25^bc^	11.56^a^	1.32	<0.01	<0.01	<0.01
S+ P	16.7^a^	18.9^ab^	21.9^b^	21.6^b^	35.0^c^	1.76	<0.01	<0.01	<0.01
Ethanol	0.33^b^	0.44^b^	0.30^b^	0.32^b^	1.09^a^	0.10	<0.01	<0.01	<0.01
Propanol	0.10^b^	0.11^b^	0.09^b^	0.09^b^	0.18^a^	0.01	<0.01	<0.01	<0.01
Ethanol + propanol	0.43^b^	0.55^b^	0.38^b^	0.41^b^	1.27^a^	0.11	<0.01	<0.01	<0.01
	Short-chain fatty acids, other acids and alcohols, mM								
Acetate	32.6^c^	34.2^bc^	38.2^abc^	43.2^ab^	45.7^a^	3.87	<0.01	<0.01	0.60
Butyrate	4.9^c^	6.0^bc^	7.3^b^	10.0^a^	13.2^a^	1.12	<0.01	<0.01	0.09
Propionate	8.1^d^	10.0^c^	13.3^b^	15.5^b^	23.7^a^	1.95	<0.01	<0.01	0.06
Caproate	0.12^c^	0.19^b^	0.20^b^	0.39^a^	0.61^a^	0.10	<0.01	<0.01	0.12
Valerate	0.47^d^	0.63^c^	0.66^bc^	0.88^b^	2.22^a^	0.22	<0.01	<0.01	<0.01
Iso-butyrate	0.95^a^	0.25^ab^	0.76^bc^	0.63^c^	0.31^d^	0.07	<0.01	<0.01	<0.01
Iso-valerate	0.47^d^	0.63^c^	0.66^bc^	0.88^b^	2.22^a^	0.22	<0.01	<0.01	0.24
Succinate	0.01^c^	0.02^c^	0.27^b^	0.19^b^	10.77^a^	1.22	<0.01	<0.01	<0.01
Methanol	0.11^c^	0.11^c^	0.14^c^	0.29^b^	5.33^a^	0.61	<0.01	<0.01	<0.01
Ethanol	0.16^b^	0.25^b^	0.17^b^	0.22^b^	1.07^a^	0.09	<0.01	<0.01	<0.01
Propanol	0.05^b^	0.06^b^	0.05^b^	0.06^b^	0.19^c^	0.02	<0.01	<0.01	<0.01
Available H_2_ and H_2_/glucose equivalent fermented									
A:P	4.1^a^	3.5^b^	2.9^c^	2.9^c^	2.1^d^	0.18	<0.01	<0.01	0.72
AB:PV	4.4^a^	3.9^b^	3.3^c^	3.3^c^	2.9^d^	0.21	<0.01	<0.01	0.82
Available H_2_, mM	66.5	69.8	77.0	90.4	81.7	8.63	0.06	0.02	0.58
GEF, mM	26.0^d^	29.1^cd^	34.3^bc^	41.1^b^	56.9^a^	3.91	<0.01	<0.01	0.02
H_2_/GEF	2.6^a^	2.4^b^	2.3^c^	2.2^c^	1.5^d^	0.08	<0.01	<0.01	<0.01
Net O/R state	−20.2^a^	−25.2	31.0^bc^	−40.2^cd^	−50.9^d^	5.04	<0.01	<0.01	0.33

FR0, 100% ryegrass; FR25, 75% ryegrass + 25% forage rape; FR50, 50% ryegrass +50% forage rape; FR75, 25% ryegrass + 75% forage rape; FR100, 100% forage rape.

NH_4_: ammonia; SCFA includes acetate, butyrate, propionate, caproate, valerate, iso-butyrate, and iso-valerate; TOFP, total organic fermentation products, includes SCFA + ethanol+ propanol+ formate + lactate + succinate; formate and lactate were detected in one sample; A:P, acetate/propionate ratio; AB:PV, (acetate+butyrate)/(propionate+valerate) ratio; GEF, glucose equivalent fermented; H_2_/GEF, available H_2_ per unit of glucose equivalent fermented; O/R state, net oxidation reduction state of rumen metabolites (excluding iso-butyrate, iso-valerate, and methanol).

SED, average standard error of the differences of means.

Treatment means within a column with different superscript are significantly different (*P <* 0.05).

Total alcohols (ethanol + propanol) as a proportion of TOFP (*P <* 0.01), and the proportions ethanol (*P <* 0.01) and propanol (*P <* 0.01) increased non-linearly with increasing forage rape inclusion in the diet (Table 3). The proportions of both ethanol and propanol were at least 3.6 and 2 times greater in FR100 than in any other dietary treatment (*P <* 0.01). The methanol concentration also increased quadratically with forage rape inclusion (*P <* 0.01) and was over 18.4 times greater in FR100 than for any other treatment.

**Figure 2.**
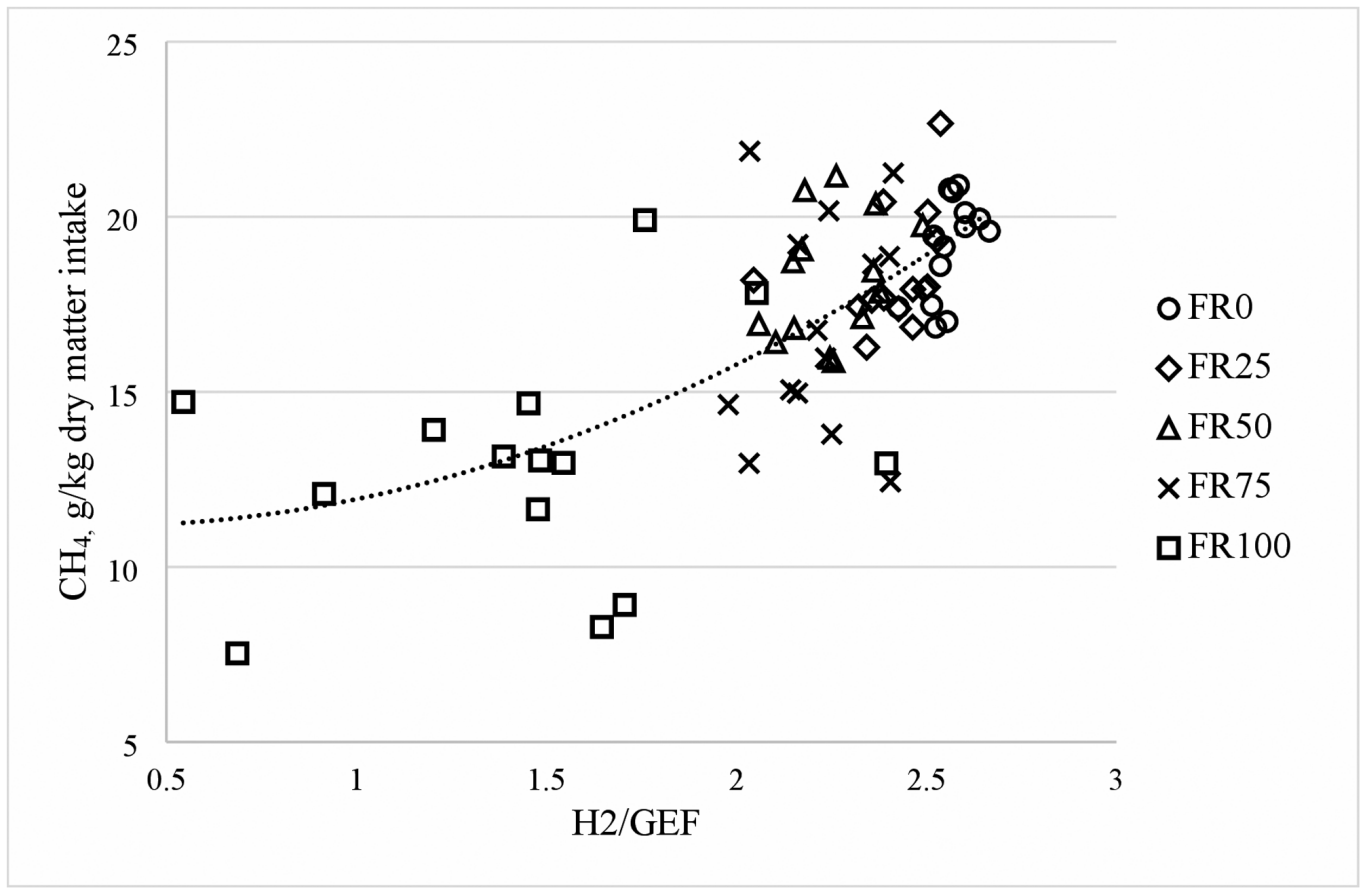
Quadratic relationship between methane (CH_4_) per unit of dry matter intake matter intake and hydrogen available per unit of glucose equivalent fermented digestibility (H2/GEF) in sheep fed ryegrass-based pasture substituted with increasing levels of forage rape: FR0, 100% ryegrass; FR25, 75% ryegrass + 25% forage rape; FR50, 50% ryegrass +50% forage rape; FR75, 25% ryegrass + 75% forage rape; FR100, 100% forage rape.

Available H_2_ produced in the rumen fluid tended to be greater in FR75 and FR100 compared with any of the other three treatments (*P =* 0.06) and increased linearly with forage rape inclusion in the diet (*P =* 0.02; Table 3). Calculated moles of glucose equivalent fermented increased nonlinearly with increasing forage rape inclusion in the diet (*P =* 0.02). The H_2_ available per unit of glucose equivalent fermented changed non-linearly with increasing forage rape inclusion in the diet (*P <* 0.01). The FR100 treatment had the lowest production of H_2_ per mol of glucose equivalent fermented (1.45), whereas FR75 and FR50 led to lower production of H_2_ per unit of glucose equivalent fermented than FR0 and FR25, and FR25 produced fewer moles of H_2_ per unit of glucose equivalent fermented than FR0. H_2_ produced per unit of glucose equivalent fermented, in a linear and quadratic regression, explained 47% of variability in CH_4_/DMI (y = 1.62 x^2^ − 1.01 x + 11.33, Figure 2) and 60% of variability in CH_4_/dDMI (y = 4.58 x^2^ − 8.388 x + 15.528). Also, H_2_ produced per unit of glucose equivalent fermented was correlated negatively with DMD (*r* = −0.81, Table 4).

The net oxidation/reduction state of the fermentation products decreased linearly (*P <* 0.01) with increasing forage rape inclusion in the diet (Table 3). pH in rumen fluid included in a linear and quadratic regression explained 65% of variability in oxidation/reduction state (y = −17.35x^2^ + 257.37x – 976.56, *R*^2^ = 0.67).

## Discussion

The hypothesis that increasing substitution of ryegrass-based pasture with forage rape in the diet of sheep would result in a linear reduction in CH_4_/DMI was not proven. Instead, CH_4_/DMI decreased quadratically with increasing forage rape inclusion in the diet, with minor numerical reduction of CH_4_/DMI in sheep fed FR25 and FR50 and more pronounced decreases as forage rape inclusion level increased from FR75 to FR100. This quadratic response was different to the previous findings of Sun et al. (2015b), who reported a linear decrease in CH_4_/DMI with increasing substitution of ryegrass with forage rape in the diet of sheep. In both the current study and the study of Sun et al. (2015b), there was a decrease in CH_4_/DMI for sheep fed FR75 and FR100 compared to those fed FR0. In the study of Sun et al. (2015b), feeding sheep FR100 resulted in a much lower CH_4_/DMI (8.2 g/kg DMI) compared to the current study (12.7 g/kg DMI) and also compared to other studies feeding FR100 where CH_4_/DMI ranged from 13.6 to 17.8 g/kg DMI (Sun et al. 2015b). Furthermore, the reduction in CH_4_/DMI between sheep fed 100% forage rape and 0% forage rape was also much greater, with 64%, in the study of Sun et al (2015b) than the 30% reduction observed in the current study. When the treatment provides a strong mitigation as for FR100 in the study of Sun et al. (2015b), there is mathematically a larger scope for a linear reduction in CH_4_/DMI. The reason for the very low CH_4_/DMI for sheep fed FR100 in the study of Sun et al. (2015b) is not known, but it may be due to seasonal differences in forage rape composition that are not yet understood.

### Factors that might explain differences in methane yield

The predicted DMD (via measured iNDF) increased linearly with increasing forage rape inclusion in the diet and CH_4_/DMI decreased quadratically with increasing DMD. A similar quadratic response in CH_4_/DMI with increasing diet digestibility has been observed when forage was substituted with increasing levels of concentrates (Moss et al., 1995, Sauvant and Noziere 2016; Jonker et al., 2016). The DMD values predicted using the iNDF method in the current study were high compared to those observed in previous total fecal collection sheep studies feeding forage rape (80%–82% DMD) and pasture (65%–75% DMD) (Sun et al. 2012, 2015a). The difference in DMD could be the result of the methodology used (iNDF in this study vs. total fecal collection).

The nonlinear response in CH_4_/DMI with increasing substitution of ryegrass with forage rape was associated with a nonlinear decrease in rumen fluid pH, less available H_2_ per mol of glucose equivalent fermented, a more reduced state of rumen fermentation products (the net oxidation/reduction state), and a nonlinear increase in actual H_2_ emissions. It has been hypothesized that the effect of forage rape on CH_4_ emissions was, at least partially, explained by a decreased rumen fluid pH when feeding forage rape compared to those fed ryegrass (Sun et al. 2015a, 2020). This larger rumen fluid pH drop is likely due to the greater content of readily fermentable carbohydrates in forage rape than in ryegrass. NRC (2001) recommends that a diet should contain a minimum of 250 g/kg DM of NDF and a maximum 440 g/kg DM of NFC to maintain normal rumen function. In the current study, the proportions of these feed components exceeded these thresholds when feeding FR75 and FR100 and these were also the two treatments with a lower ruminal pH. The decrease in rumen fluid pH could result in reduced CH_4_ emissions because rumen methanogens are sensitive to a low pH (Van Kessel and Russell, 1996; Janssen, 2010). This likely leads to a greater concentration of dissolved H_2_ in the rumen liquid (i.e., the dissolved H_2_ pool) due to decreased H_2_ use by methanogens. This, in turn, leads to less formation of H_2_ (flux through the pool) by fermenting microbes due to thermodynamic inhibition and a compensatory change to the formation of more reduced organic end products (Janssen, 2010). This might therefore partially explain why CH_4_/DMI was lower in sheep fed FR75 and FR100. Rumen fluid pH was positively related with changes in rumen fermentation that result in less H_2_ production, i.e., the available H_2_ available per glucose equivalent fermented, and the net oxidation/reduction state of fermentation products decreased when pH decreased as well.

Increased dissolved H_2_ in the rumen fluid are also expected to lead to increased exhaled H_2_ by the animal (Wang et al., 2013), explaining the observed increase in H_2_ emissions from the sheep. Sheep fed FR100 emitted 0.37 g H_2_/kg DMI, which theoretically would have reduced the CH_4_/DMI by about 7% relative to sheep fed FR0. Sheep fed FR75 (and also FR50) emitted 0.12 g H_2_/kg DMI, which would have reduced the CH_4_/DMI by about 1.5% relative to sheep fed FR0. The H_2_ emissions per unit of DMI were previously reported to range from 0.026 (Sun et al., 2015a) to 2.52 g/kg (Sun et al., 2020) for sheep fed FR100, but these H_2_ emissions do not fully explain the reductions in CH_4_.

### Presence of seldom rumen fermentation products

The difference between the theoretical CH_4_/DMI reductions accounted for the H_2_ emission and those observed require a fuller description of H_2_ balances per unit of glucose fermented by examining intermediaries not often described in studies of rumen fermentation. In the current study, sheep on FR100 had the most distinct rumen fermentation profile with (as % of TOFP) more than 26.3 times greater succinate, 3.6 times greater ethanol, 1.8 times greater valerate, 1.6 times greater propanol, and 0.3 times less acetate than in any of the other treatments. All these changes in aggregate translate to 0.4 times less available H_2_ per unit of glucose equivalent fermented. Succinate is a precursor for propionate and is an electron sink that consumes the same number of electrons as propionate. Succinate normally does not accumulate in rumen fluid (Kennedy et al., 1991), but high concentrations were measured in FR100. The pathway catalyzing the transformation of succinate to propionate requires the cobalt-containing coenzyme B_12_ (Nagaraja et al., 1997), and accumulation of succinate was found in the rumens of sheep fed a cobalt deficient diet (Kennedy et al., 1991). Therefore, the lower cobalt concentration in the forage rape than in ryegrass may have led to succinate accumulation in FR100. Rumen methanogens also have a requirement for cobalt (Thauer et al., 1993; Keltjens and Vogels, 1993). If there was a cobalt limitation at high inclusion levels of forage rape that limited CH_4_ formation by methanogens, and simultaneously there was an increased requirement for cobalt for propionate formation, this could explain the quadratic response of CH_4_/DMI with increasing forage rape inclusion in the diet.

The percentages of butyrate, valerate, and caproate were greater in both FR75 and FR100 compared to other treatments. Electrons are consumed during the synthesis of these three metabolites and their formation is likely promoted to deal with the potential excess of dissolved H_2_ in the rumen fluid, as an alternative to using electrons for H_2_ formation (Ungerfeld, 2015) when forage rape inclusion in the diet is increased. The increase in the proportions of ethanol and propanol in the rumen fluid of sheep fed FR100 compared with the other treatments also suggests that they were synthesized as electron acceptors to deal with the increased dissolved H_2_ in the rumen environment (Czerkawski and Breckenridge, 1972). Accumulation of ethanol was previously observed in the rumen of animals fed rapidly fermentable feed (Allison et al., 1964).

These changes in rumen fermentation profiles led to the measured rumen fluid metabolites of sheep fed FR100, FR75, FR50, and FR25 being 2.5, 2.0, 1.5, and 1.2 times more reduced, respectively, than in sheep fed FR0. Also, compared to FR0, the calculated theoretical amount of H_2_ (available H_2_) released per mol of glucose equivalent fermented was 0.42, 0.15, 0.12, and 0.08 times less for FR100, FR75, FR50, and FR25. These calculations show that electrons from fermentation were diverted to producing reduced fermentation products instead of being used to generate H_2_. Also, the available H_2_ per fermented glucose explained a greater proportion of the variability in CH_4_/DMI and CH_4_/dDMI than previous SCFA ratios reported in Jonker et al. (2016) or equations based on multiple SCFAs in Williams et al. (2019). The effect of lowering pH and the assumed increased rumen dissolved H_2_ concentration, inferred by the H_2_ exhaled (Wang et al., 2013), are both linked to a faster fermentation rate in forage rape, which might drive the redirection of electrons towards more reduced fermentation products at the expense of producing more H_2_.

### Presence of methanol in rumen liquid

Forage rape contains a significant amount of pectin. Hydrolysis of methoxy groups present in pectins by rumen microbes resulted in the release of methanol. The concentration of methanol in the rumen fluid of sheep fed FR100 was almost 48 times greater (and 2.6 times greater in FR75) than in sheep fed FR0, whereas the methanol concentration was similar in sheep fed FR25 and FR50 to those fed FR0. This suggests that the methanol from forage rape pectins was largely used by methylotrophic methanogens in sheep fed FR25, FR50, and FR75, but less so in sheep fed FR100. Methanol also accumulated in vitro when forage rape was incubated with a low strength buffer (resulting in reduced pH at the end of incubation), but not when forage rape was incubated with a high strength buffer (Della Rosa et al., 2021). These results suggest that the methylotrophic archaea might also be sensitive to a low pH, that methanol utilization is less efficient at a low pH, or that these were also affected by low cobalt availability.

## Conclusions

Increasing the proportion of forage rape in the diet of lambs resulted in a quadratic decrease in CH4/DMI; FR100 and FR75 reduced CH4/DMI by 31% and 11%, respectively, compared with FR0, with smaller reductions with FR25 and FR50. The FR75 and especially FR100 treatments also resulted in a lower rumen pH than in FR0. The low pH might be due to the high concentration of rapidly fermentable nonfiber carbohydrates in forage rape, which consequently likely was the driver for the shift in rumen fermentation pathways toward the increased production of more reduced fermentation products such as propionate, succinate, ethanol, propanol, valerate, and caproate. Methanol was also present in the rumen fluid of sheep fed FR100, suggesting that methanol utilization by methylotrophic methanogens was impaired when animals were fed FR100, but not when fed FR25, FR50, and FR75. The contribution of potential cobalt limitation to reduced CH_4_/DMI is unknown, but it could have played a role.

## Supplementary Material

skac223_suppl_Supplementary_Table_S1

## Abbreviations

ADF: acid detergent fiber
CH_4_: methane
CH_4_/DMI: g methane per kg of dry matter intake
CH_4_/dDMI: g methane emission per kg of dry matter digested
CO_2_: carbon dioxide
Cfat: crude fat
CP: crude protein
DMD: dry matter digestibility
DMI: dry matter intake
FR0: 100% ryegrass
FR25: 75% ryegrass + 25% forage rape
FR50: 50% ryegrass +50% forage rape
FR75: 25% ryegrass + 75% forage rape
FR100: 100% forage rape
GEF: glucose equivalent fermented
H_2_: hydrogen
iNDF: indigestible neutral detergent fiber
NFC: nonfiber carbohydrates
NDF: neutral detergent fiber
SCFA: short-chain fatty acids
TOFP: total organic fermentation products
