# Supplementary material for: Substituting ryegrass-based pasture with graded levels of forage rape in the diet of lambs decreases methane emissions and increases propionate, succinate, and primary alcohols in the rumen
Source: J Anim Sci. 2022 Jun 20;100(9):skac223. doi: 10.1093/jas/skac223 (PMC9486902; doi:10.1093/jas/skac223)
Supplement: skac223_suppl_Supplementary_Table_S1 [file skac223_suppl_supplementary_table_s1.docx]

|  |  | Activities |  |
| --- | --- | --- | --- |
| Day |  |  | |
| ***Paddocks/Pens*** | | | |
| 1 - 9 |  | Grazing ryegrass (n = 20) and forage rape for 8, 16, 24 h for 3 days at each stage (n = 80) | |
| 10-21 |  | Grazing ryegrass (n = 20) and forage rape (n =80) for 24 h per day | |
| 22-36 |  | Sheep moved into 10 group pens (2 pens/treatment), 16 sheep/treatment (n=14 + 2 spares per treatment) | |
| ***Individual crates/chambers*** | | | |
| 37-38 |  | Measurement chamber batch 1 in crates  29 sheep in crates (measurement sheep + 1 spare per treatment), 5 sheep for FR0, all other treatments 6 sheep/treatment.  DMI records, faeces sampling. | |
| 39-40 |  | Measurement batch 1 in chambers  24 sheep in chambers, 4 sheep for FR0, all other treatments 5 sheep/treatment.  DMI records, faeces sampling, | |
| 41 |  | Rumen and blood sampling after exiting chambers – 18 h after the last feed delivery- (i.e. pre feeding). | |
| 44-45 |  | Measurement batch 2 in crates  29 sheep in crates (measurement sheep + 1 spare per treatment), 5 sheep for FR25, all other treatments 6 sheep/treatment.  DMI records, faeces sampling. | |
| 46-47 |  | Measurement batch 2 in chambers  24 sheep in crates, 4 sheep for FR25, all other treatments 5 sheep/treatment.  DMI records, faeces sampling | |
| 48 |  | Rumen and blood sampling after exiting chambers – 18 h after the last feed delivery (i.e. pre feeding). | |
| 51-52 |  | Measurement batch 3 in crates  27 sheep in crates (measurement sheep + 1 spare per treatment), 6 sheep for FR0% and FR25, all other treatments 5 sheep/treatment.  DMI records, faeces sampling. | |
| 53-54 |  | Measurement batch 3 in chambers  22 sheep in crates, 5 sheep for FR0 and FR25, all other treatments 4 sheep/treatment.  DMI records, faeces sampling. | |
| 55 |  | Rumen and blood sampling after exiting chambers – 18 h after the last feed delivery (i.e. pre feeding). | |
| Day 1 was June 15^th^2020. Treatments: 100% ryegrass (FR0), 75% ryegrass + 25% forage rape (FR25), 50% ryegrass +50% forage rape (FR50), 25% ryegrass + 75% forage rape (FR75 and 100% forage rape (FR100), DMI: dry matter intake | | |  |

Table S1. Schedule of diet transition and adaptation and measurement phases
